# Nonrigid Registration of Lung CT Images Based on Tissue Features

**DOI:** 10.1155/2013/834192

**Published:** 2013-11-14

**Authors:** Rui Zhang, Wu Zhou, Yanjie Li, Shaode Yu, Yaoqin Xie

**Affiliations:** ^1^Key Laboratory of Health Informatics, Shenzhen Institutes of Advanced Technology, Chinese Academy of Sciences, 1068 Xueyuan Avenue, Shenzhen 518055, China; ^2^Harbin Institute of Technology Shenzhen Graduate School, Shenzhen 518055, China

## Abstract

Nonrigid image registration is a prerequisite for various medical image process and analysis applications. Much effort has been devoted to thoracic image registration due to breathing motion. Recently, scale-invariant feature transform (SIFT) has been used in medical image registration and obtained promising results. However, SIFT is apt to detect blob features. Blobs key points are generally detected in smooth areas which may contain few diagnostic points. In general, diagnostic points used in medical image are often vessel crossing points, vascular endpoints, and tissue boundary points, which provide abundant information about vessels and can reflect the motion of lungs accurately. These points generally have high gradients as opposed to blob key points and can be detected by Harris. In this work, we proposed a hybrid feature detection method which can detect tissue features of lungs effectively based on Harris and SIFT. In addition, a novel method which can remove mismatched landmarks is also proposed. A series of thoracic CT images are tested by using the proposed algorithm, and the quantitative and qualitative evaluations show that our method is statistically significantly better than conventional SIFT method especially in the case of large deformation of lungs during respiration.

## 1. Introduction

Lung cancer is the most common cause of cancer-related death all over the world, with exceeding 1 million deaths annually [1]. Image-guided radiation therapy (IGRT) plays an important role in both the curative and palliative treatment of lung cancer, and precise targeting of lung tumors is an essential step in IGRT [2]. However, it is difficult to target tumors in lungs by taking into account respiration and tumor motion. Deformable or nonrigid image registration has been recognized as a key technology to locate position of tumor precisely [3]. By definition, image registration is a process of establishing spatial correspondences between two images. It can be classified into rigid registration and nonrigid registration. As the motion and shape change of lung is nonlinear, it is appropriate to register the lung CT images by using nonrigid registration.

Various nonrigid image registration methods have been applied for the lung CT images. In general, the registration methods can be divided into intensity-based and feature-based methods. There are a lot of intensity-based methods used in the thoracic CT image registration, such as fast intensity-based freeform registration [4], multiresolution optical flow technique [5], regional narrow shell model [6], and demons algorithm [7]. However, intensity-based methods tend to misregister small structures in the lung like vessels and airways as they only rely on image intensity [8]. Feature-based methods are typically applied when the local structural information is distinctive and salient, and the performance of feature-based registration is closely related to the accuracy of feature extraction and matching in images. 

In feature-based registration methods which were commonly used in thoracic images, both Rohr [9] and Coselmon et al. [10] used manually extracted landmarks and an approximating thin-plate spline to describe the mapping function. However, manual landmark extraction often takes much time and is tedious, and the accuracy greatly depends on the experience of doctors. Han [2] proposed a hybrid method that used features detected by Forstner operator as constrains to guide an intensity-based deformable registration. Gorbunova et al. [11] combined intensity, curves, and surfaces to register lung CT images. These methods were hybrid techniques that combined feature-based and intensity-based methods. Recently, Xie et al. [12] and Urschler et al. [8] showed the feature-based registration based on scale-invariant feature transform (SIFT) [13] and thin-plate spline (TPS) achieved promising results in thoracic CT images. The robustness of SIFT has been properly evaluated, which shows well performance and stability under arbitrary affine transformations for MR, CT, and ultrasound images due to its distinctive and superior advantages in feature detection and description [14, 15]. However, the accuracy of extracted key points and the matching strategy have not been fully explored. Our work mainly focuses on these two questions.

In the present work, we develop a hybrid feature extraction method that can detect anatomic tissue features of lungs effectively based on Harris and Stephens [16] and SIFT. To effectively remove the mismatched features, a novel method based on cross-correlation and structural invariance is also proposed. 

## 2. Methods

 The proposed method consists of four major steps: tissue feature detection, feature description, feature matching and mismatched points removing, and thin-plate spline (TPS) transformation [17]. We will illustrate the method in the following sections in detail. As the voxel spacings in three directions are different, we first preprocess the images into a volume with isotropic spacing. This process can improve computation speed and reduce memory usage due to the increase of voxel spacing of each slice. In addition, having an isotropic voxel spacing can increase the accuracy of SIFT descriptor.

### 2.1. Tissue Feature Detection

 The SIFT algorithm detects key points of extremes in the scale space, and the detected key points are blob features which cannot fully reflect the movement of lungs. The features that we need to find are lung boundaries, vessel bifurcations, and alveoli. These features usually have high intensity gradient; thus we use Harris algorithm which can find points with high intensity gradient.

 The Harris detector is based on the local autocorrelation function of a signal, which measures the local changes of the signal with patches shifted by a small amount in different directions [18]. In 3D images, the local autocorrelation is defined as

(1) c(x,y,z) =∑W[I(xi+Δx,yi+Δy,zi+Δz)−I(xi,yi,zi)]2,

where *I*(·, ·, ·) denotes the image function and (*x*_*i*_, *y*_*i*_,*z*_*i*_) are the points in the Gaussian window *W* centered on (*x*, *y*, *z*).

 The shifted image can be approximated by a Taylor expansion truncated to the first order terms:

(2) c(x,y,z) =[ΔxΔyΔz]  ×[∑xi,yi,ziW·Ix2∑xi,yi,ziW·IxIy∑xi,yi,ziW·IxIz∑xi,yi,ziW·IxIy∑xi,yi,ziW·Iy2∑xi,yi,ziW·IyIz∑xi,yi,ziW·IxIz∑xi,yi,ziW·IyIz∑xi,yi,ziW·Iz2]  ×[ΔxΔyΔz],

where  *I*_*x*_, *I*_*y*_, and *I*_*z*_ denote the intensity gradient in *x*-, *y*-, and *z*-axises. 

 The eigenvalues of the matrix contain enough local information related to the neighborhood structure, and points with three high eigenvalues are selected as the features. To reduce computational complexity and get a good distribution of feature points in the lungs, the strategy we used is to select the key points which have three high eigenvalues and each key point has a distance larger than a threshold with others.

### 2.2. Local SIFT Feature Descriptor

**Figure 1:**
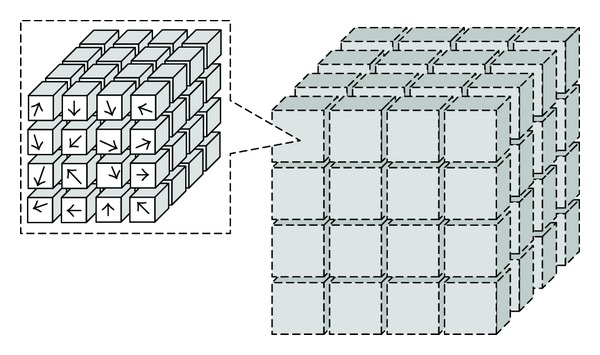
The 3D SIFT descriptor covers a 16 × 16 × 16 voxel region, the whole region divided into 4 × 4 × 4 subregions. For each subregion, 8 × 4 bins histogram is calculated to summarize the gradient orientation of 4 × 4 × 4 voxels in the subregion.

 The SIFT descriptor is robust to local deformations and to errors in feature detection. It is considered as one of the most effective descriptors currently available [19]. For each detected feature, a distinctive local SIFT feature descriptor is built [20, 21]. The 3D SIFT descriptor is characterized by using the gradient orientation distribution in a 16 × 16 × 16 grid surrounding the feature position, and the cube is divided into 4 × 4 × 4 subregions. The descriptor structure is shown in Figure 1. 

Each voxel has two values which represent the direction of the gradient in three dimensions. One is from 0° to 360°, and the other is from −90° to 90°. To build the orientation histograms, each bin indicates 45°. Thus each subregion has an 8 × 4 bins histogram to summarize the gradients of the voxel in it. Therefore, a total of 2048 vectors are calculated for a given feature.

### 2.3. Feature Matching and Removing Mismatched Points

In the process of feature matching, the best candidate match for each key point is found by identifying its nearest neighbor in the dataset of key points from images. The nearest neighbors are defined as the key points with minimum Euclidean distance from the given descriptor vector. The probability that a match is correct can be determined by taking the ratio of distance from the closest neighbor to the distance of the second closest [13]. To find corresponding feature points, we first convert the histograms of a feature to a single vector and compare the *l* ^2^ distance between a feature vector in template image against every feature vector of the target image. Suppose *S*_1_ and *S*_2_ are the best match (which has the lowest distance) and the second best match of feature *S*, and *d*_1_, *d*_2_ are the corresponding distance between features  *S*_1_, *S*_2_ with *S*. If the ratio *r* = *d*_1_/*d*_2_ is below a threshold *τ*, then  *S*_1_ is chosen tentatively as the corresponding feature of *S*. The threshold *τ* Lowe [13] rejected all matches in which the distance ratio *τ* is greater than 0.8, which eliminates 90% of the false matches while discarding less than 5% of the correct matches. In our tests, we set *τ* to be 0.8 experientially. To further improve the accuracy of above vector matching, we perform the matching process twice by reversing the roles of the two volumes and consider only those matches as valid for which the features are still matched from volume *V*_1_ to *V*_2_ and from *V*_2_ to *V*_1_. However, there are still many wrong matched pairs after the above process.

 To remove these mismatched points, we proposed a novel method based on cross-correlation and structural invariance for the matching verification. Cross-correlation is a similarity metric between two signals. We can remove those mismatches which have slightly large differences in the neighborhood areas around candidate corresponding key points by cross-correlation. The correlation coefficient of two corresponding features is calculated according to

(3) $$\begin{array}{c} \text{CC}=\frac{\sum_{\left(i,j,k\right)}\left(A\left(i,j,k\right)-\overset{-}{A}\right)\left(B\left(i,j,k\right)-\overset{-}{B}\right)}{\sqrt{\sum_{\left(i,j,k\right)}{\left(A\left(i,j,k\right)-\overset{-}{A}\right)}^{2}}\sqrt{\sum_{\left(i,j,k\right)}{\left(B\left(i,j,k\right)-\overset{-}{B}\right)}^{2}}}, \end{array}$$

where (*i*, *j*, *k*) are the coordinates of voxels within a distance *d* surrounding the features position, *A* and *B* are the intensity values of corresponding voxel, and $\overset{-}{A}$ and $\overset{-}{B}$ are the mean intensity values. These intensity values are weighted by a Gaussian window and normalized. If the correlation coefficient CC is larger than a threshold, we deem that they are similar to each other. We experientially set d to be 5 voxels. 

 Even by using cross-correlation, there are still some mismatched points. As we know, the relative position of structures in lungs would not change too much during respiration. Therefore, if there are a number of key points surrounding a feature in the lung during inspiration, these key points would remain surrounding this feature during expiration. The approach based on structural invariance consists of two steps. First, a number of points are selected which have the shortest distance to the tested feature in an image. Second, the same number of points are selected which are nearest to the corresponding feature in the other image. If the number of the matched point pairs is larger than a given threshold, we affirm that the tested features are corresponding. This process is illustrated in Algorithm 1.

**Figure 2:**
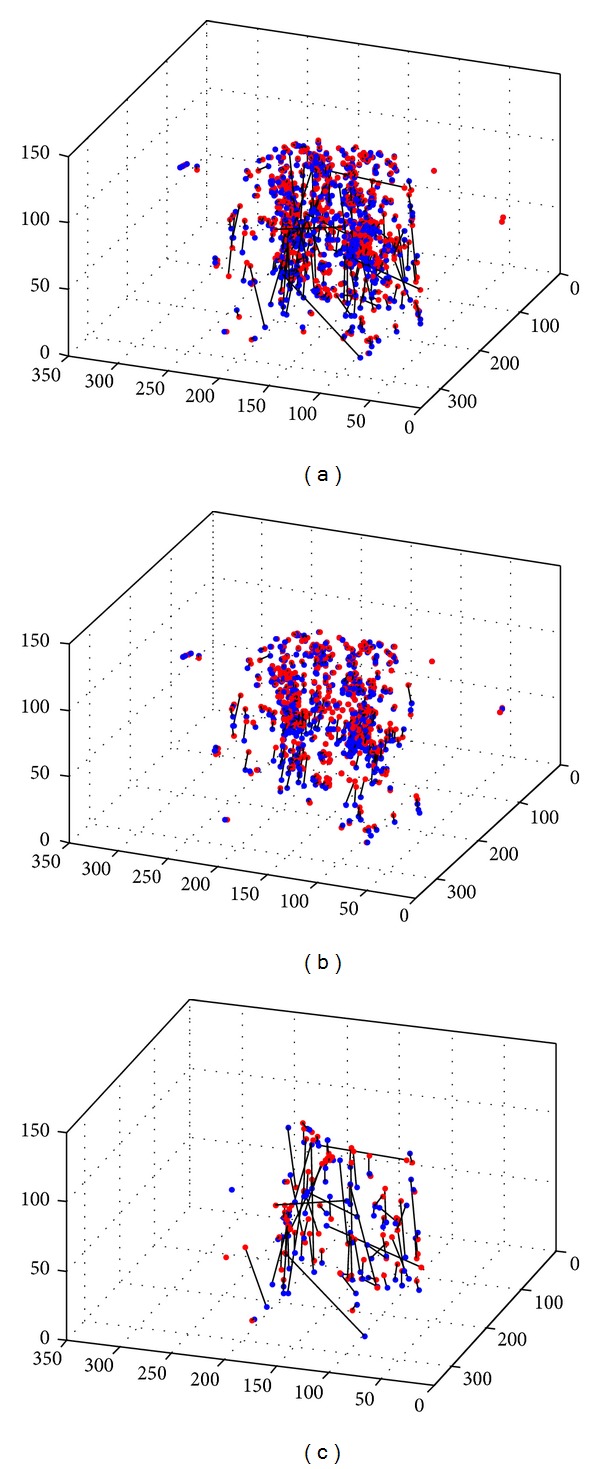
(a) The matched pairs only using symmetrical nearest neighborhood method. (b) The retained pairs after using our method. (c) The removed point pairs using our method. The red points are points detected in one thoracic volume, and the blue points are in the other. The black lines link matched pairs.

Figure 2 shows the result before and after using the above method. These control point pairs are detected using Harris detector and intensity thresholds are used to insure the main points detected are in the region of lungs; we can see that the above method can remove mismatched point pairs effectively.  Table 1 shows the number of key point pairs in the process of matching verification using cross correlation and structural invariance.

### 2.4. Thin-Plate Spline Transformation

The process of nonrigid registration warps the template image to the target image in a way that they can best match on a voxel-by-voxel basis. Mathematically, this is an optimization problem, in which a set of transformation parameters transform the voxels in the template image to their corresponding voxels in the target image [22]. 

To find the transformation matrix that maps an arbitrary voxel on the template image to that on the target image, TPS transformation is employed in this study. This method can be used to establish voxel-to-voxel correspondence of a region of interest according to the paired control points. A detailed description of the TPS method can be found in the studies by Bookstein [17]. 

## 3. Results and Discussion

The performance of the above method was evaluated by a series of thoracic CT images. Each image had 80 slices, and was reconstructed with a 2.5 mm slice thickness. Each CT slice was discretized into 512 × 512 voxels, and the voxel spacing is 0.977 mm. All the images are handled on a personal computer with Pentium 2.8 GHz Dual-Core and 3 GB memory, and the proposed methods are implemented using C++. Insight Segmentation and Registration Toolkit (ITk) [23] is also used. Some programs are referred to Xie et al. [12] and Cheung and Hamarneh [21]. The mean of the square sum of intensity differences (SSD) and image intensity cross-correlation coefficient (CC) is used to evaluate the quality of the registration images quantitatively.

**Figure 3:**
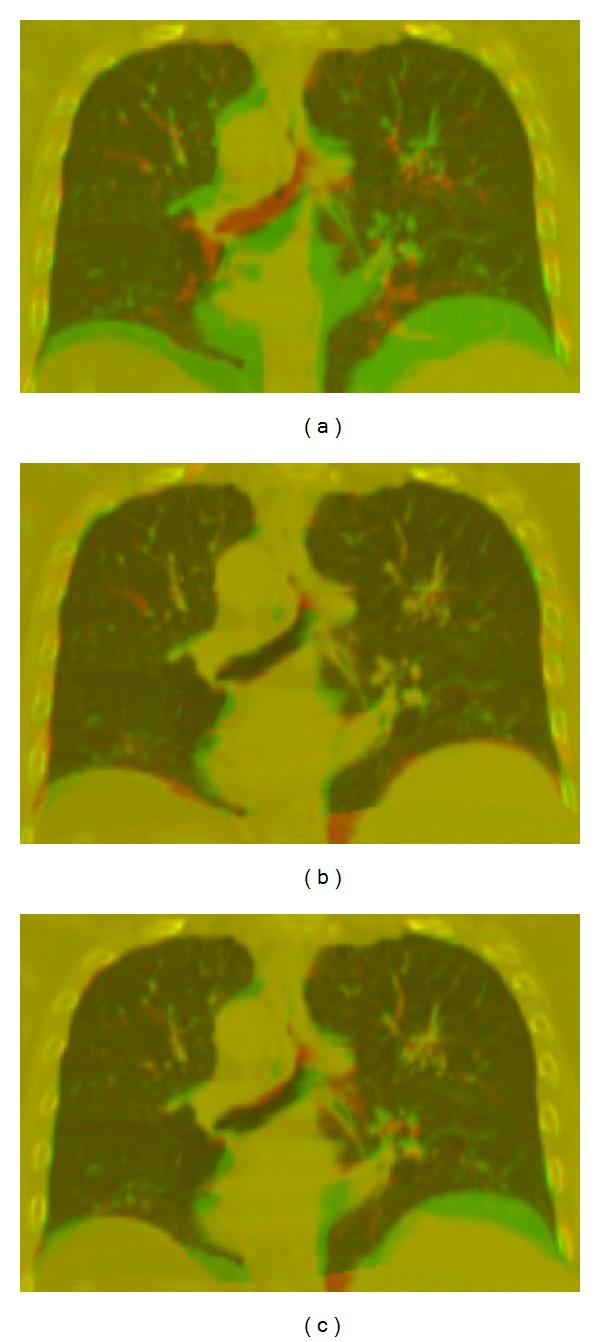
Fusion images of two phases of digital phantom: (a) before registration, (b) after registration using the proposed method, and (c) after registration using the conventional SIFT method.

Figure 3(a) shows the fusion image of the template and target phases before registration.  Figure 3(b) shows the fusion image of the two phases using the proposed approach. For comparison, the results of the conventional SIFT approach are also shown in Figure 3(c). The red region stands for the target image, and the green region stands for the template image.

To be quantitative, we listed the results of these two methods in Table 2. It is clear that by using the proposed method, SSD was reduced from 56727 to 37650, and the CC was increased from 0.646 to 0.740, demonstrating that the proposed method is much better than the conventional SIFT.

We also choose 15 points to reflect the average errors of the above registration methods. The results are listed in Table 3. The errors are computed by point coordinates in target images minus the point coordinates in register images. On average, using our hybrid method, the mean absolute deviation in three directions of the 15 points was reduced from 1.6 to 0.6 mm, from 1.9 to 0.7 mm, and from 2.8 to 0.7 mm, respectively. The standard deviation (SD) in three directions of the 15 points was reduced from 2.6 to 0.9 mm, from 2.2 to 1.0 mm, and from 5.2 to 1.2 mm, respectively. From the data in Table 3, it is clear that the proposed method is more accurate than conventional SIFT.

**Figure 4:**
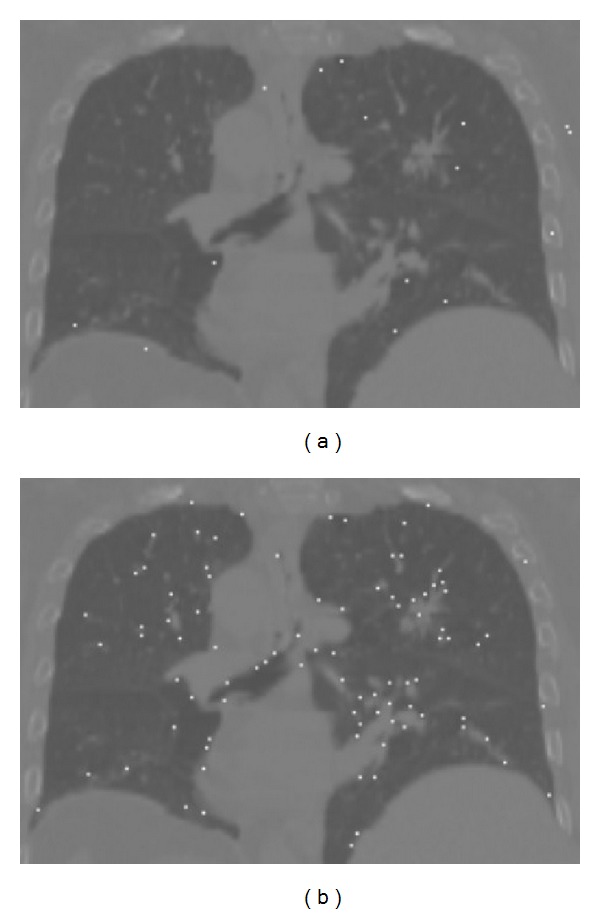
Feature point detection: (a) SIFT, (b) proposed method. The white dots stand for the detected feature points.

Generally, the registration results by using TPS method depend on the number and locations of the control points. In our work, as we only use an intensity threshold rather than a true segmentation to preprocess the volume, the detected points by using SIFT were spread in the phantom, only a small part of them was located on the lungs. However, the detected points by using proposed method were mostly on the lungs. Both of these two methods got about 500 matched point pairs (SIFT: 539 pairs, proposed method: 490 pairs). Though the matched point pairs by using the proposed method were fewer than those by using SIFT, the detected points on the lungs by using the proposed method were statistically significantly more than those by using SIFT.  Figure 4 shows results of detected feature points using these two methods.

## 4. Conclusions

In the present work, a hybrid feature-based nonrigid image registration method is proposed. It can effectively detect the tissue features. Promising results were obtained using clinical thoracic data. Furthermore, our proposed method to remove mismatched points is automatic and robust. It can guarantee the correctness of point pairs and can be a significant supplementary of former feature-based registration methods.

## Figures and Tables

**Algorithm 1:**
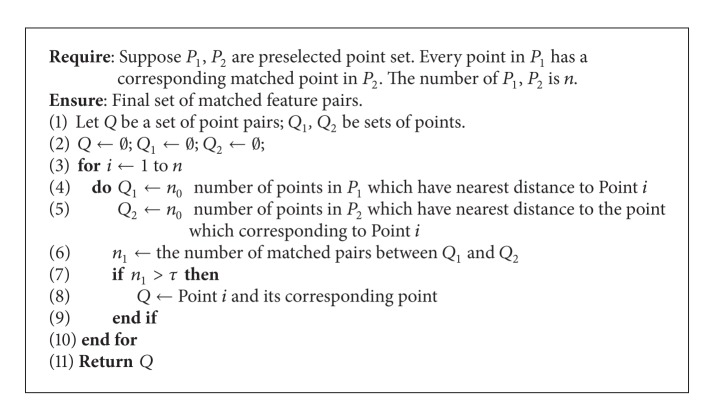
Structural invariance removing method.

**Table 1:** The number of key point pairs in the process of matching verification.

Candidate matching pairs	Removed by cross-correlation	Removed by structural invariance	Retained number
625	47	88	490

**Table 2:** Comparison of the proposed method and the conventional SIFT.

Assessment	Before registration	Conventional SIFT	Proposed method
SSD	104151	56727	37650
CC	0.472	0.646	0.740

**Table 3:** Errors of 15 representative points using the proposed and conventional SIFT.

Point index	Point coordinates in template image (mm)	Point coordinates in target image (mm)	Errors of conventional SIFT (mm)	Errors of proposed method (mm)
1	191.6, 224.7, 21.0	191.6, 219.8, 40.5	7.1, −1.9, 10.5	2.2, 2.4, 3.0
2	294.6, 234.3, 21.0	299.5, 227.3, 51.0	4.2, −4.2, 18.0	0.7, 1.4, 3.0
3	287.6, 265.1, 45.0	288.3, 260.9, 64.5	1.6, 1.4, 0.0	0.7, 0.0, 0.0
4	189.4, 246.9, 55.5	186.0, 242.0, 72.0	−0.1, −2.1, 1.5	0.6, 0.0, 0.0
5	309.2, 299.1, 54.0	305.7, 299.8, 70.5	−1.4, 0.7, 1.5	−0.7, −0.7, 0.0
6	284.1, 353.3, 82.5	280.7, 351.2, 82.5	−0.6, 0.6, −1.5	−1.0, −0.4, −1.5
7	188.8, 297.0, 81.0	184.7, 295.6, 90.0	1.4, 2.1, 0.0	0.0, −0.7, 0.0
8	280.7, 266.4, 73.5	279.3, 265.0, 90.0	−0.7, 3.5, 3.0	0.0, 0.0, 1.5
9	289.7, 306.0, 93.0	286.2, 303.9, 100.5	−0.7, −0.7, 0.0	0.0, −0.7, 0.0
10	188.8, 236.5, 96.0	186.1, 233.7, 106.5	−2.7, −2.1, 6.0	−0.7, −0.7, 1.5
11	309.9, 303.3, 106.5	305.7, 304.0, 111.0	−3.5, 1.4, 0.0	−0.7, −0.6, 0.0
12	191.6, 279.6, 109.5	187.5, 278.9, 114.0	0.1, 1.4, 0.0	0.1, 0.7, 0.0
13	262.6, 346.4, 117.0	257.7, 341.5, 117.0	0.0, −3.4, 0.0	2.1, 0.0, 0.0
14	190.9, 271.9, 123.0	190.2, 269.9, 123.0	0.0, −0.7, 0.0	0.0, −0.7, 0.0
15	319.6, 263.6, 135.0	318.9, 269.9, 139.5	0.0, 2.1, 0.0	0.0, −1.4, 0.0
